# Supplementary material for: Halogen-Based 17β-HSD1 Inhibitors: Insights from DFT, Docking, and Molecular Dynamics Simulation Studies
Source: Molecules. 2022 Jun 20;27(12):3962. doi: 10.3390/molecules27123962 (PMC9229637; doi:10.3390/molecules27123962)
Supplement: Supplementary file 1 [file molecules-27-03962-s001.zip › molecules-1732159-supplementary.pdf]

molecules

Supplementary Materials

Halogen-Based 17β-HSD1 Inhibitors: Insights from DFT, Docking, and Molecular Dynamics Simulation Studies

Arulsamy Kulandaisamy [1,*,†], Murugesan Panneerselvam [2,†], Rajadurai Vijay Solomon [3,*], Madhavan Jaccob [2,*], Jaganathan Ramakrishnan [4], Kumaradhas Poomani [4], Muralikannan Maruthamuthu [5] and Nagendran Tharmalingam [6]

1 Department of Biotechnology, Bhupat and Jyoti Mehta School of Biosciences, Indian Institute of Technology Madras, Chennai 600 036, Tamil Nadu, India
2 Department of Chemistry and Computational Chemistry Laboratory, Loyola Institute of Frontier Energy, Loyola College, Chennai 600 034, Tamil Nadu, India; panneerchem130491@gmail.com
3 Department of Chemistry, Madras Christian College (Autonomous), Tambaram East, Chennai 600 045, Tamil Nadu, India
4 Laboratory of BioCrystallography and Computational Molecular Biology, Department of Physics, Periyar University, Salem 636 011, Tamil Nadu, India; rjaganphy@gmail.com (J.R.); kumaradhas@yahoo.com (K.P.)
5 Division of Pharmacoengineering and Molecular Pharmaceutics, Eshelman School of Pharmacy, University of North Carolina, Chapel Hill, NC 27599, USA; murali.kbiotech@gmail.com
6 Division of Infectious Diseases, Rhode Island Hospital, Alpert Medical School, Brown University, Providence, RI 02903, USA; micronagu@gmail.com
* Correspondence: bt15d045@smail.iitm.ac.in (A.K); vjsolo@mcc.edu.in (R.V.S.); jaccob@loyolacollege.edu (M.J.)
† These authors contributed equally to this work.

Table S1. Quantum chemical descriptors (in eV) of newly designed inhibitors.

Molecules	*S*	*η*	*μ*	*ω*	E_g
**Ref**	**-0.24**	**2.12**	**-3.39**	**2.72**	4.24
$_{F}$**R1**	-0.24	2.12	-3.49	2.88	4.24
$_{F}$**R2**	-0.23	2.17	-3.62	3.03	4.33
$_{F}$**R3**	-0.24	2.12	-3.52	2.92	4.24
$_{F}$**R4**	-0.23	2.18	-3.85	3.41	4.35
$_{Cl}$**R1**	-0.25	1.99	-3.63	3.31	3.98
$_{Cl}$**R2**	-0.23	2.20	-3.71	3.14	4.39
$_{Cl}$**R3**	-0.24	2.12	-3.57	3.00	4.24
$_{Cl}$**R4**	-0.23	2.20	-3.99	3.62	4.40
$_{Br}$**R1**	-0.29	1.74	-3.87	4.30	3.47
$_{Br}$**R2**	-0.23	2.17	-3.75	3.24	4.33
$_{Br}$**R3**	-0.24	2.12	-3.56	3.00	4.24
$_{Br}$**R4**	-0.26	1.95	-4.22	4.57	3.91
$_{I}$**R1**	-0.32	1.54	-4.06	5.36	3.08
$_{I}$**R2**	-0.23	2.17	-3.76	3.26	4.34
$_{I}$**R3**	-0.24	2.11	-3.59	3.05	4.23
$_{I}$**R4**	-0.29	1.73	-4.49	5.83	3.46

Table S2. The binding energy/docking score of newly designed halogen-based inhibitors against with 17β-HSD1 receptor.

Molecules	Binding free energy (Kcal/mol)	Binding affinity (Ki)
**Reference (R)**	-10.21	32.98 nM
$_{F}$**R1**	-10.5	20.16 nM
$_{F}$**R2**	-10.29	28.43 nM
$_{F}$**R3**	-10.26	32.71 nM
$_{F}$**R4**	-10.53	19.05 nM
$_{Cl}$**R1**	-10.86	10.91 nM
$_{Cl}$**R2**	-10.82	11.73 nM
$_{Cl}$**R3**	-10.72	13.85 nM
$_{Cl}$**R4**	-11.58	3.27 nM
$_{Br}$**R1**	-11.46	3.99 nM
$_{Br}$**R2**	-11.23	5.89 nM
$_{Br}$**R3**	-11.01	8.46 nM
$_{Br}$**R4**	-11.71	2.59 nM
$_{I}$**R1**	-11.69	2.68 nM
$_{I}$**R2**	-11.4	4.40 nM
$_{I}$**R3**	-11.29	5.27 nM
$_{I}$**R4**	-11.94	1.78 nM

Table S3. The detailed information on hydrogen bond and π-bond interactions between 17β-HSD1 receptor and halogen-based inhibitors.

Molecules	H-bond Interacting Residues	No of Interactions	π -bond Interacting Residues	No of Interactions
$_F$**R**$_1$	Gly 141, Lys159, His221	3	Val143, Leu149, Cys185, Pro187, His221, Phe226	6
$_F$**R**$_2$	Ser142, Cys185, His221, Glu282	4	Val143, Met147, Leu149, Pro187, Val225, Phe259	9
$_F$**R**$_3$	Ile14, Gly15, Thr140, Gly144	5	Val143, Cys185, Pro187, Val188	6
$_F$**R**$_4$	Gly186, Val188, His221	3	Val143, Met147, Leu149, Tyr155, Cys185, Pro187, Val225, Phe226	11
$_{Cl}$**R**$_1$	Gly141, Lys159, His221	3	Val143, Leu149, Cys185, Pro187, Phe226, Phe259	6
$_{Cl}$**R**$_2$	Val188, His221	2	Val143, Leu149, Cys185, Gly186, Pro187, Ala191, Lys195, Val225, Phe226	11
$_{Cl}$**R**$_3$	Gly186, Val188	2	Val143, Leu149, Pro187, Ala191, Lys195, Val225, Phe226, Phe259, Met279	10
$_{Cl}$**R**$_4$	Gly186, Pro187, Val188	3	Val143, Leu149, Cys185, Pro187, Lys195, His221, Val225, Phe226, Phe259	15
$_{Br}$**R**$_1$	Gly141, Asn152, Lys159, His221	4	Val143, Leu149, Tyr155, Cys185, Lys195, His221, Phe259	7
$_{Br}$**R**$_2$	Glu194, Tyr218, His221, Ser222	4	Val143, Gly186, Pro187, Glu194, Lys195, His221, Val225, Phe259	12
$_{Br}$**R**$_3$	Gly186, Val188	2	Val143, Leu149, Pro187, Ala191, Lys195, His221, Val225, Phe226, Phe259, Met279	11
$_{Br}$**R**$_4$	His221	1	Val143, Tyr155, Gly186, Pro187, Lys195, His221, Val225, Phe259	13
$_I$**R**$_1$	Gly141, Asn152, Tyr218, His221	4	Val143, Leu149, Tyr155, Cys185, Pro187, Val188, His221, Phe259	10
$_I$**R**$_2$	Val188, His221, Ser222	3	Val143, Leu149, Cys185, Pro187, Ala191, Lys195, Val225, Phe226	9
$_I$**R**$_3$	Gly186, Val188	2	Val143, Leu149, Pro187, Ala191, Lys195, His221, Val225, Phe226, Phe259, Met279	12
$_I$**R**$_4$	Val188	1	Leu149, Cys185, Pro187, Lys195, Val225, Phe226, Phe259	8

Table S4. The 17β-HSD1 receptor residues are involved in the halogen bond interactions with halogen-based inhibitors.

Molecules	Halogen Bond Interacting Residues	No of Interactions
$_{F}$**R**$_1$	Leu149, Met279, Leu262, Val143	7
$_{F}$**R**$_2$	His221, Ser222, Val225, Met279, Tyr218, Val283	9
$_{F}$**R**$_3$	Val143, Leu149, Gly144	9
$_{F}$**R**$_4$	Val225, Val188, Ile14, Lys195, His221, Met279, Phe259, Glu194, Phe226, Tyr155	22
$_{Cl}$**R**$_1$	Leu149, Leu262, Met279, Phe259	8
$_{Cl}$**R**$_2$	Ser142, Cys185, Tyr155	4
$_{Cl}$**R**$_3$	Met279, Phe259, His221, Leu149, Leu262, Val225	8
$_{Cl}$**R**$_4$	Val225, Ile14, Val188, Lys195, His221, Phe259, Glu194, Phe226, Tyr155	18
$_{Br}$**R**$_1$	Leu149, Met279, His221, Ser222, Tyr218, Val283	11
$_{Br}$**R**$_2$	Val143, Gly186, Pro187	6
$_{Br}$**R**$_3$	Met279, Phe259, Glu282, His221, Leu262, Val225	8
$_{Br}$**R**$_4$	Leu149, Val143, Met279, Tyr155, Lys159, Pro150, Tyr218, Gly186, Phe259, Pro187	19
$_{I}$**R**$_1$	His221, Met279, Ser222, Leu149, Tyr218, Val225, Val283	10
$_{I}$**R**$_2$	Cys185, Ser142, Tyr155	5
$_{I}$**R**$_3$	Glu282, Met279, Phe259, Val225, His221	9
$_{I}$**R**$_4$	Ala191, Leu149, Met279, Phe259, Val225, Cys185, Ser142, Tyr155	13
